# Heat shock protein 90 regulates necroptosis by modulating multiple signaling effectors

**DOI:** 10.1038/cddis.2016.25

**Published:** 2016-03-03

**Authors:** C K Yang, S D He

**Affiliations:** 1 Cyrus Tang Hematology Center and Collaborative Innovation Center of Hematology, Jiangsu Key Laboratory of Preventive and Translational Medicine for Geriatric Diseases, Soochow University, Suzhou, China; 2 Jiangsu Institute of Hematology, the First Affiliated Hospital of Soochow University, Suzhou, China

Necroptosis has recently been identified as a form of programmed necrosis.^1^ It is characterized morphologically by membrane rupture and organelle swelling. Emerging evidence has demonstrated that receptor-interacting kinase 1 (RIP1 or RIPK1),^2^ RIP3 (or RIPK3),^3^ and pseudokinase mixed lineage kinase domain-like protein (MLKL)^4^ are the core components of this process. Necroptosis is induced by various initiators, including the TNF family death receptors, the Toll-like receptors, IFNRs, and certain pathogens.^5^ Among these inducers, the cytokine TNFα is the most extensively studied trigger of necroptosis. In TNFα-induced necroptosis, RIP1 and RIP3 form a protein complex, termed the necrosome,^3^ through the RIP homotypic interaction motif (RHIM) of both proteins, leading to phosphorylation and activation of RIP3. Then, activated RIP3 phosphorylates its substrate MLKL. Upon phosphorylation, MLKL forms oligomers and translocates to the plasma membrane to induce necrotic cell death.^6, 7, 8^ In addition, RHIM-containing proteins such as TRIF, DAI, and ICP6 are known to activate RIP3.^5^ Thus, RIP3 is considered to be a central signaling molecule for programmed necrosis; it receives upstream signals and further transduces necrosis signals to MLKL. Although multiple studies have determined the molecular basis of the activation of RIP3 and MLKL, the exact, finely tuned molecular mechanism for regulating RIP3 and MLKL activation has not been fully elucidated. To this end, two new studies by Jacobsen *et al.*^9^ and Zhao *et al.*^10^ published in the issue of *Cell Death and Disease*, along with recent work by Li *et al.,*^11^ reveal that heat shock protein 90 (HSP90) regulates the stability and function of RIP3 and MLKL.

HSP90 has been characterized as a molecular chaperon that modulates both the structure and function of associated proteins referred to as clients. It is known that numerous kinases and pseudokinases are HSP90 clients, and these proteins form complexes with HSP90 and its co-chaperone CDC37.^12^ Loss of HSP90 function likely causes the destabilization and degradation of its clients via the ubiquitin–proteasome pathway. A previous study has demonstrated RIP1 as an HSP90 client.^13^ Inhibition of HSP90 function disrupted association between HSP90 and RIP1, and resulted in the degradation of RIP1.^13^ Further, loss of HSP90 activity blocked TNF-induced RIP1-dependent NF-κB activation and necrosis, and made cells sensitive to TNF-induced apoptosis.^13, 14^ Therefore, HSP90 is a chaperone protein that is required to maintain the stability and function of RIP1 in the necroptosis pathway.

As RIP3 is well established as a key kinase regulating necroptosis, it is of interest to determine whether or not RIP3 is a client of HSP90. Recently, Li *et al.*^11^ identified HSP90 and CDC37 as RIP3-associated proteins and dissected the essential role of the HSP90-CDC37 complex in RIP3 activation. Addition of the HSP90 inhibitor 17AAG disrupted the association of RIP3 with HSP90, but this interaction was not affected by CDC37 RNAi. Further, both inhibition of HSP90 and knockdown of CDC37 blocked the formation of the RIP1-RIP3 necrosome, the phosphorylation of RIP3, and necroptosis. Remarkably, polymerized RIP3-induced necrosis, in which RIP1 is not required, was efficiently blocked by disruption of HSP90 function. Therefore, HSP90 is able to regulate necroptosis by directly modulating RIP3 activation.

It has become clear that the functions of RIP1 and RIP3 in necroptosis are modulated by HSP90. Little is known about the regulation of MLKL function. A fascinating observation was made by both the Murphy^9^ and Zhang^10^ research groups: HSP90 modulates MLKL stability and function in the necroptosis pathway. Jacobsen *et al.*^9^ found that seven HSP90 inhibitors in a small-molecule library that they screened provided protection against necrotic death driven by the auto-activating mutant form of MLKL Q345A. Consistently, treatment with HSP90 inhibitors or knockdown of CDC37 blocked the death of *Rip3*^−/−^*Mlkl*^−/−^ fibroblasts expressing the mutant form of MLKL S345D, which mimics the RIP3-mediated phosphorylation of MLKL. Zhao *et al.*^10^ identified HSP90 as an MLKL-associated protein using yeast two-hybrid screening. Overexpression of HSP90 enhanced MLKL-induced cell death in 293T cells lacking RIP3 expression. These findings demonstrate that HSP90 is a modulator of MLKL-mediated cell death that functions either directly on MLKL or downstream of MLKL. MLKL oligomerization and membrane translocation are essential events in necroptosis. Of note, inhibition of HSP90 function prevented the oligomerization of mutant MLKL S345D and its membrane translocation.^9^ In contrast, overexpression of HSP90 enhanced MLKL oligomerization and increased the amount of MLKL translocation to the membrane.^10^ Thus, HSP90 activity is essential for the assembly of MLKL into oligomers and the translocation of these oligomers to the membrane, supporting a regulatory role for HSP90 in necroptosis by directly modulating MLKL function.

Another interesting observation is that the levels of RIP3 and MLKL decreased significantly following prolonged treatment with HSP90 inhibitors.^9, 10, 11, 15^ Such degradation of RIP3 or MLKL was prevented by the proteasome inhibitor MG132.^10, 11^ Although HSP90 appears to have a role in the maintenance of RIP3 and MLKL stability, the levels of these two proteins were not markedly affected following incubation with HSP90 inhibitors,^9, 11^ even when a reduced RIP1 level was observed.^9^ These observations suggest that suppression of necroptosis by HSP90 inhibitors is not likely to be caused by a delayed reduction in the RIP3 or MLKL level. Indeed, short-term inhibition of HSP90 seems to cause a conformational change in RIP3,^11^ indicating that HSP90 is required to maintain both the functional conformation of RIP3 and the activation of RIP3. Given that HSP90 is essential for the oligomerization of MLKL and its membrane translocation,^9, 10^ HSP90 may be required to facilitate the transition of MLKL to an active conformation that enables the subsequent trafficking of MLKL to the plasma membrane. Thus, HSP90 may have a crucial role in modulating the functions of RIP3 and MLKL in the necroptosis pathway, rather than simply controlling their stability.

Recent studies have indicated that the impact of HSP90 on necroptosis varies remarkably among species and cell type. In line with this idea, treatment with 17AAG was shown to completely prevent TNF-induced necroptosis in both human cells and rat primary macrophages, but this inhibitor failed to affect necroptosis in mouse primary macrophages.^11^ Interestingly, there was no detectable interaction between HSP90 and RIP3 in mouse primary macrophages.^11^ Addition of 17AAG or other HSP90 inhibitors protected mouse fibroblasts from necroptosis,^9, 15^ although these inhibitors were more toxic to mouse fibroblasts than they were to human cells.^9^ Therefore, HSP90 inhibitors provide better protection against necroptosis in human and rat cells than in mouse cells. These observations raise the question of whether or not HSP90 inhibitors are able to repress necroptosis-associated injury *in vivo*. Notably, Li *et al.*^11^ provided intriguing evidence that injection of an HSP90 inhibitor attenuated TNF-induced systemic inflammatory response syndrome in rats. This finding has significant implications for potential treatments for necroptosis-associated diseases with HSP90 inhibitors.

In conclusion, these three papers illustrate how HSP90 regulates necroptosis by modulating the stability and function of core necroptosis regulators RIP3 and MLKL. These findings, together with the previous discovery that HSP90 is involved in regulating RIP1, establish that HSP90 has an extremely important role in tightly governing necroptosis by directly modulating the following three critical signaling effectors of the necroptosis pathway: RIP1, RIP3, and MLKL (Figure 1). Increasing evidence is indicating that necroptosis contributes to the pathogenesis of many human diseases, including inflammatory, neurodegenerative, and autoimmune diseases.^1^ The intervention in necroptosis with HSP90 inhibitors could represent a novel strategy for the treatment of necroptosis-related diseases.

## Figures and Tables

**Figure 1:**
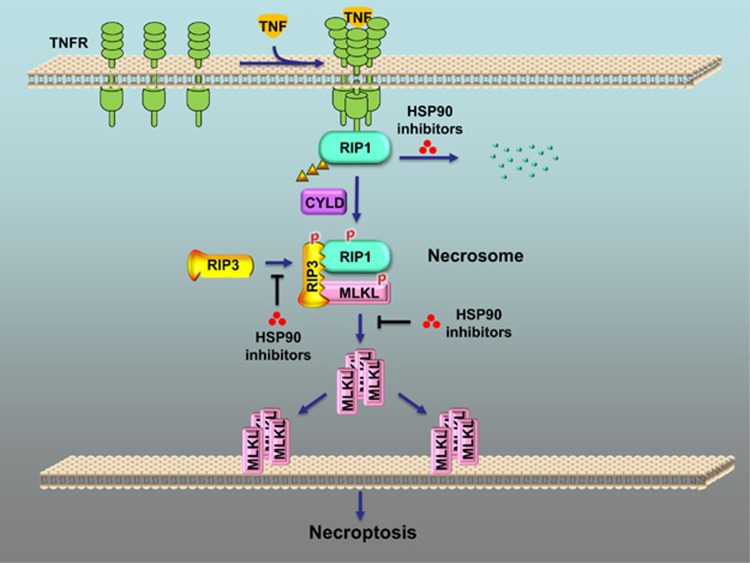
The role of HSP90 in the regulation of necroptosis. In TNF-induced necroptosis, RIP1 is deubiquitinated by cylindromatosis (CYLD) and then binds to RIP3 to form a necrosome, leading to the activation of RIP3. Subsequently, activated RIP3 phosphorylates MLKL. Phosphorylated MLKL forms oligomers and translocates to the plasma membrane, inducing necroptosis. Inhibition of HSP90 function blocks necroptosis by directly disrupting the following steps: (i) RIP1 stability, (ii) RIP3 activation, and (iii) MLKL oligomerization and translocation to the membrane
